# Time Course of Gene Expression of Inflammatory Mediators in Rat Lung after Diesel Exhaust Particle Exposure

**DOI:** 10.1289/ehp.7696

**Published:** 2005-02-10

**Authors:** K. Murali Krishna Rao, Jane Y. C. Ma, Terence Meighan, Mark W. Barger, Donna Pack, Val Vallyathan

**Affiliations:** Pathology and Physiology Research Branch, Health Effects Laboratory Division, National Institute for Occupational Safety and Health, Morgantown, West Virginia, USA

**Keywords:** chemokines, diesel, lung, molecular biology, monocyte/macrophage

## Abstract

Diesel exhaust particles (DEPs) at three concentrations (5, 35, and 50 mg/kg body weight) were instilled into rats intratracheally. We studied gene expression at 1, 7, and 30 days postexposure in cells obtained by bronchoalveolar lavage (BAL) and in lung tissue. Using real-time reverse transcriptase-polymerase chain reaction (RT-PCR), we measured the mRNA levels of eight genes [interleukin (*IL*)*-1*β, *IL-6*, *IL-10*, *iNOS* (inducible nitric oxide synthase), *MCP-1* (monocyte chemoattractant protein-1), *MIP-2* (macrophage inflammatory protein-2), *TGF*-β*1* (transforming growth factor-β1), and *TNF-*α (tumor necrosis factor-α)] in BAL cells and four genes [*IL-6*, *ICAM-1* (intercellular adhesion molecule-1), *GM-CSF* (granulocyte/macrophage-colony stimulating factor), and *RANTES* (regulated upon activation normal T cell expressed and secreted)] in lung tissue. In BAL cells on day 1, high-dose exposure induced a significant up-regulation of IL-1β, iNOS, MCP-1, and MIP-2 but no change in IL-6, IL-10, TGF-β1, and TNF-α mRNA levels. There was no change in the mRNA levels of IL-6, RANTES, ICAM-1, and GM-CSF in lung tissue. Nitric oxide production and levels of MCP-1 and MIP-2 were increased in the 24-hr culture media of alveolar macrophages (AMs) obtained on day 1. IL-6, MCP-1, and MIP-2 levels were also elevated in the BAL fluid. BAL fluid also showed increases in albumin and lactate dehydrogenase. The cellular content in BAL fluid increased at all doses and at all time periods, mainly due to an increase in polymorphonuclear leukocytes. *In vitro* studies in AMs and cultured lung fibroblasts showed that lung fibroblasts are a significant source of IL-6 and MCP-1 in the lung.

Diesel exhaust particles (DEPs) are generated by heavy-duty diesel engines used in several industries and motor vehicles used in public transportation. They are ultrafine respirable particles with an average diameter of < 2.5 μm and contain several mutagenic and carcinogenic hydrocarbons (Arlt et al. 2003). The adverse health effects resulting from exposure to DEPs are well recognized (McClellan 1987; Sydbom et al. 2001). Epidemiologic studies have shown an increased risk of respiratory morbidity and mortality associated with exposure to DEPs (Salvi 2001; Schwartz 1994).

Exposure to DEPs has been shown to cause adverse reactions in the lungs (Diaz-Sanchez et al. 1994) and other tissues (Yoshino et al. 2002). Several studies have shown that the phagocytic activity of macrophages is suppressed exposure to DEPs (Castranova et al. 1985; Prasad et al. 1988). DEP exposure *in vitro* or *in vivo* also affects lipopolysaccharide-induced production of cytokines [tumor necrosis factor-α (TNF-α) and interleukin-1 (IL-1)] in alveolar macrophages (AMs) (Yang et al. 1997, 1999). Similarly, DEP exposure also affects production of cytokines in the lung epithelial cells (Bayram et al. 1998; Steerenberg et al. 1998).

The production of cytokines involves transcriptional activation of the genes. The aim of the present study was to investigate the mRNA levels of several genes that have been implicated in inflammatory response in AMs and lung tissue at various time points after intratracheal instillation of DEPs in rats, and to correlate these findings with cytokine production in AMs and cultured lung fibroblasts exposed *in vitro* to DEPs. The genes involved include those for cytokines, such as *IL-1*β (Goodman et al. 1982), *IL-10* (Huaux et al. 1998), *TNF-*α (Dubois et al. 1989), and transforming growth factor (*TGF*; Williams et al. 1993; Williams and Saffiotti 1995), and chemokines, such as monocyte chemoattractant protein-1 (*MCP-1*; Barrett et al. 1999) and macrophage inflammatory protein-2 (*MIP-2*; Driscoll et al. 1993). Also involved are the nonprotein inflammatory mediator nitric oxide, generated mainly through inducible nitric oxide synthase (iNOS; Castranova et al. 1998), and adhesion molecules, such as intercellular adhesion molecule-1 (ICAM-1; Hubbard and Giardina 2000; Nario and Hubbard 1996). In addition, granulocyte/macrophage-colony stimulating factor (GM-CSF) is purported to play an important role in numerous respiratory illnesses, including asthma (Xing et al. 1996). It is generated by a variety of lung cell types (Bergmann et al. 2000; Blau et al. 1994; Christensen et al. 2000; Churchill et al. 1992; Fitzgerald et al. 2003; O’Brien et al. 1998; Smith et al. 1990; Soloperto et al. 1991). RANTES (regulated upon activation normal T cell expressed and secreted) is another cytokine that has been implicated in lung inflammatory responses (Johnston et al. 1998).

Most of the studies cited above have been performed under different contexts. The advent of real-time reverse transcriptase–polymerase chain reaction (RT-PCR) methods makes it possible to study the expression of several genes implicated in the inflammatory response under identical conditions to evaluate the time course of expression of these genes. Therefore, we studied the expression of the mRNA levels of several of these cytokines and correlated these observations with the inflammatory response as assessed by measuring the influx of cells and protein into the bronchoalveolar space. Further, cytokine levels were measured in bronchoalveolar lavage (BAL) fluid. The results show that DEPs up-regulate several genes implicated in the inflammatory response, at both the message and protein levels, within 24 hr in cells obtained by BAL, representing both polymorphonuclear neutrophils (PMNs) and AMs. To elucidate the role of interactions between different cell populations in the production of inflammatory mediators in the lung, we also performed *in vitro* Transwell co-culture experiments using AMs and lung fibroblasts.

## Materials and Methods

### Animals.

The animals used in these experiments were specific pathogen-free Sprague-Dawley rats [HLA:(SD)CVF; Hilltop Laboratories, Scottdale, PA], weighing about 175 g. The animals were housed in an environmentally controlled facility accredited by the Association for Assessment and Accreditation of Laboratory Animal Care. The rats were monitored to be free of endogenous viral pathogens, parasites, mycoplasmas, *Helicobacter*, and ciliary-associated respiratory bacillus. Rats were acclimated for at least 5 days before use and were housed in ventilated cages provided with HEPA-filtered air; Alpha-Dri virgin cellulose chips (Shepherd Specialty Papers, Watertown, TN) and hardwood Beta chips (NEPCO, Warrensburg, NY) were used as bedding. The rats were maintained on 2018S Teklad Global 18% Rodent Diet (Harlan Teklad, Madison, WI) and tap water, both of which were provided *ad libitum*.

### Reagents.

DEPs with an average mass median diameter of 0.5 μm were obtained from standardized heavy-duty diesel engine emission (sample 2975; National Institute of Standards and Technology, Gaithersburg, MD). We obtained cytokine kits (rat) for MCP-1 and MIP-2 from Biosource (Camarillo, CA). The culture medium for BAL cells consisted of Eagle’s minimum essential medium (BioWhittaker, Walkersville, MD), 1 mM glutamine (GIBCO, Life Technologies, Grand Island, NY), 10 mM HEPES (Sigma Chemical Company, St. Louis, MO), 100 U/mL penicillin-streptomycin (GIBCO), 100 μg/mL kanamycin (GIBCO), and 10% (vol/vol) heat-inactivated fetal bovine serum (BioWhittaker).

### *Experimental design for* in vivo *studies*.

Animals were intratracheally instilled with either saline or 5, 35, or 50 mg DEP suspension/kg body weight (low, medium, and high doses), respectively. Groups of animals (*n* = 4 per group) representing each treatment were sacrificed on study days 1, 7, and 30 to obtain BAL cells and lung tissue. We used an additional set of four control and four experimental animals to obtain more data for the 50 mg dose on day 1.

### Intratracheal instillation of DEPs.

Rats were anesthetized with an intraperitoneal injection of sodium methohexital (30–40 mg/kg body weight Brevital; Eli Lilly, Indianapolis, IN) and were intratracheally instilled using a 20-gauge 4-in. ball-tipped animal feeding needle. DEPs were suspended in endotoxin-free, Ca^2+^/Mg^2+^-free phosphate-buffered saline (PBS; BioWhittaker) and sonicated for 1 min. Rats were given 5, 35, or 50 mg DEP suspension/kg body weight or an equivalent volume of PBS.

### Isolation of AMs.

The animals were anesthetized with pentobarbital sodium (50 mg/kg body weight) and exsanguinated by cutting the abdominal aorta. Alveolar cell populations were obtained by BAL according to the method of Myrvik et al. (1961). The lungs from each animal were lavaged eight times with 5 mL phosphate-buffered medium (145 mM NaCl, 5 mM KCl, 9.4 mM Na_2_HPO_4_, and 1.9 mM NaH_2_PO_4_, pH 7.4). The cells were separated from the lavage fluid by centrifugation at 300 × *g* for 5 min and then washed three times by alternate centrifugation and resuspension in phosphate-buffered medium. The numbers of AMs and PMNs were determined according to their unique cell diameters, using an electronic cell counter equipped with a cell-sizing unit (Coulter Multisizer II with a 256C Channelizer; Coulter Electronics, Hialeah, FL). The cells were then resuspended in the culture medium for use in all experiments.

### Isolation of lung fibroblasts.

We isolated lung fibroblasts as described by Reist et al. (1991). Briefly, the lungs were perfused with normal saline and lavaged with PBS containing 0.1% glucose and sectioned four times at 0.5-mm intervals with a McIlwain tissue chopper (Campden Instruments, Lafayette, IN). The chopped lung tissue from a single rat was digested in 20 mL HEPES-buffered solution (145 mM NaCl, 5 mM KCl, 1 mM CaCl_2_, 5.5 mM glucose, and 10 mM HEPES, pH 7.4) containing collagenase (0.1%), elastase (40 U/mL), bovine serum albumin (0.5%), and DNAse (0.018%) in a shaker water bath for 30 min at 37°C. The digested mixture was filtered through two layers of sterile gauze that had been washed with culture medium. The cells were sedimented by centrifugation and plated in six-well culture plates. The medium was changed 24 hr later, and the cells were allowed to grow to confluence.

### Transwell experiments with fibroblasts and AMs.

To measure mRNA expression in separated cell populations and to study the interaction of soluble mediators released by cell populations, we conducted experiments in Transwell chambers (CoStar, Corning, NY). For these experiments, cultured lung fibroblasts were trypsinized, and 1 million cells were plated in the outer well of a Transwell plate and cultured for 24 hr. At the end of the 24-hr period, freshly isolated AMs (1 million cells) were placed in the inserts. DEPs (200 μg/mL) were added either to the macrophages in the inner wells or to the fibroblasts in the outer well and incubated for 4 hr. Total RNA was isolated from each population separately.

### Inflammatory mediators in BAL fluid.

For measurement of cytokines in the BAL fluid, the first lavage was collected, spun down to sediment cells at 300 × *g*, and the supernatant was stored at −80°C until measurements were performed. Lactate dehydrogenase (LDH) and albumin were measured within 24 hr on refrigerated samples with a COBAS MIRA Plus analyzer (Roche Diagnostics, Indianapolis, IN) using kits from Roche Diagnostics and Sigma-Aldrich (St. Louis, MO), respectively.

### Measurement of cytokines and NO production.

We measured the cytokines in BAL fluid and in culture supernatants of AMs after 24 hr of culture. IL-6, MCP-1, and MIP-2 were measured by enzyme-linked immunosorbent assay (ELISA) kits according to the manufacturer’s instructions (Biosource, Camarillo, CA). NO in the supernatants was measured as the stable oxidation product of NO, nitrite. We then measured nitrite production using the Greiss reaction (Green et al. 1982). The amount was calculated from a standard curve using sodium nitrite.

### Quantitation of mRNAs by RT-PCR.

We measured the cytokine mRNA levels using a SYBR Green PCR kit with the ABI 5700 Sequence Detector (PE Applied Biosystems, Foster City, CA). Total RNA was isolated from AMs (≈ 2 million cells) or lung tissue after BAL (≈ 50 mg wet tissue) using RNAqueous-4PCR kits (Ambion, Austin, TX). DNAse I-treated RNA (1–2 μg) was reverse transcribed using SuperScript II (Life Technologies, Gaithersburg, MD). The complementary DNA generated was diluted 1:100, and 15 μL was used to conduct the PCR reaction according to the SYBR Green PCR kit instructions. The comparative *C*_T_ (threshold cycle) method was used to calculate the relative concentrations (Applied Biosystems 1997). Briefly, the method involves obtaining the *C*_T_ values for the cytokine of interest, normalizing to a housekeeping gene (*18S* in the present case) and deriving the fold increase compared with control, unstimulated cells. The primer sets for RANTES were as follows: forward, ACT CCC TGC TGC TTT GCC TAC C; reverse, TTG GCG GTT CCT TCG AGT GAC (product, 123 base pairs). The primer sets for other genes have been published previously (Rao et al. 2004).

### Statistical methods.

To evaluate the data we used a *t*-test assuming unequal variance, or a *Z*-test for means, or a nonparametric Wilcoxon/Kruskal-Wallis test. The significance was set at *p* < 0.05.

## Results

### Markers of inflammation and inflammatory cells in BAL fluid.

**Figure 1:**
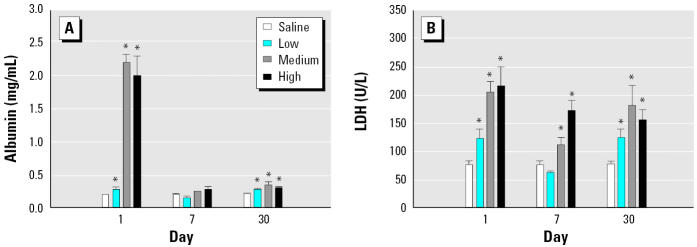
Albumin (*A*) and LDH (*B*) levels in BAL fluid (mean ± SE from at least three animals).
*Significantly greater than control, *p* < 0.05.

**Figure 2:**
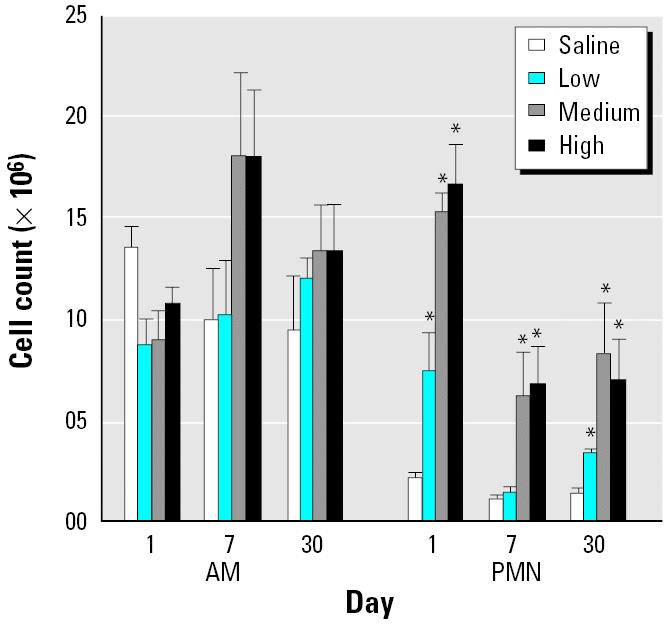
Cellular contents of BAL fluid (mean ± SE from at least three animals).
*Significantly greater than control, *p* < 0.05.

We measured the inflammatory response in the lung after DEP exposure by determining the classical inflammatory markers albumin, LDH, and cell numbers in the BAL fluid. Albumin showed significant increases on days 1 and 30 after exposure at all three DEP doses (Figure 1A). LDH levels were elevated in BAL fluid at all time points and at all doses except at the 5 mg dose on day 7 (Figure 1B). Figure 2 shows the cell numbers in BAL fluid. There was no significant increase in the number of alveolar AMs in BAL fluid under any conditions. PMNs were increased on day 1 at all three doses; the numbers remained high at the two higher doses of DEPs on day 7 and at all three doses on day 30.

### *Expression of genes of inflammatory mediators in BAL cells* in vivo.

**Figure 3:**
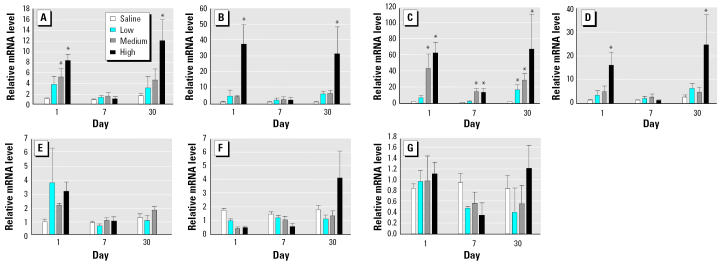
Time course of mRNA expression in lung cells obtained by BAL after DEP exposure. (*A*) IL-1β. (*B*) iNOS. (*C*) MCP-1. (*D*) MIP-2. (*E*) IL-10. (*F*) TGF-β1. (*G*) TNF-α. Bars represent fold increase above control (mean ± SE from at least three animals for each inflammatory mediator).
*Significantly greater than control, *p* < 0.05.

**Figure 4:**
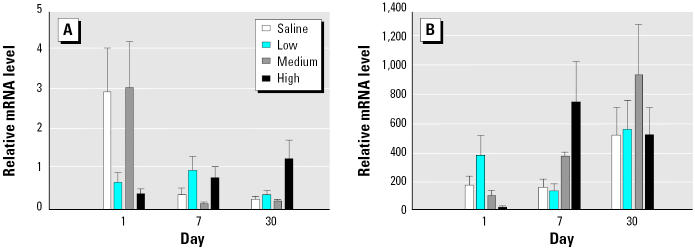
IL-6 mRNA levels in BAL cells (*A*) and lavaged lung tissue (*B*) after DEP exposure. mRNA levels in lung tissue are expressed in relation to BAL cells; bars represent mean ± SE from at least three animals.

To identify the mediators that may be involved in promoting the inflammatory response, we measured mRNA levels of eight genes in BAL cells immediately after isolation. On day 1, diesel particles at the highest dose (50 mg/kg body weight) induced significant up-regulation of IL-1β, iNOS, MCP-1, and MIP-2 in BAL cells (Figure 3A). By day 7, the mRNA levels came down at all doses, with only MCP-1 still showing significantly higher mRNA levels at medium and high doses of DEPs. On day 30 the mRNA levels of MCP-1 were significantly elevated at all three doses of DEPs, but a significant up-regulation of IL-1β, iNOS, and MIP-2 message levels was seen only at the high dose. Thus, the temporal response of these cytokines tended to be bimodal. In contrast, there was no change in IL-10, TGF-β1, or TNF-α mRNA levels in BAL cells (Figure 3B). Likewise, we noted no significant changes in IL-6 mRNA levels of BAL cells after DEP exposure (Figure 4).

### Gene expression in the lung tissue.

**Figure 5:**
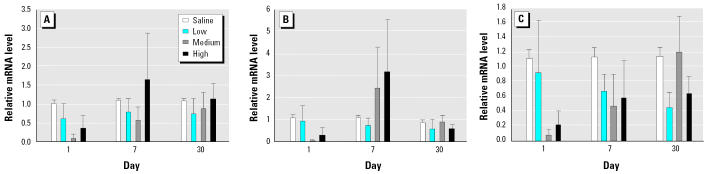
Time course of mRNA expression in lavaged lung tissue after DEP exposure. (*A*) GM-CSF. (*B*) ICAM-1. (*C*) RANTES. Bars represent fold increase above control (mean ± SE from at least three animals for each inflammatory mediator).

We isolated total RNA from the lung tissue after lung lavage and measured the mRNA levels of IL-6 (Figure 4), GM-CSF, ICAM-1, and RANTES (Figure 5). None of these showed any change in expression.

### Inflammatory mediators in the BAL fluid.

**Figure 6:**
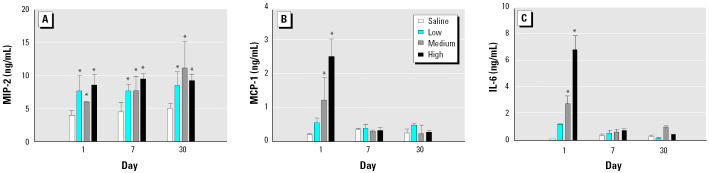
MIP-2 (*A*), MCP-1 (*B*), and IL-6 (*C*) levels in BAL fluid after DEP exposure. The bars represent mean ± SE from at least three animals.
*Significantly greater than control, *p* < 0.05.

**Figure 7:**
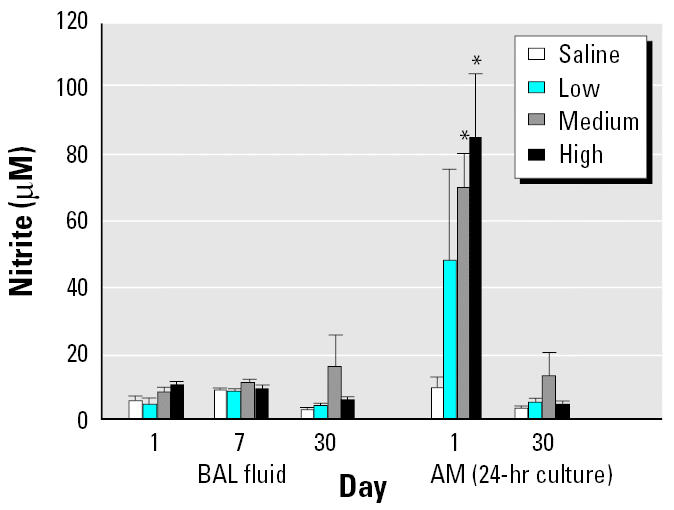
NO levels in BAL fluid and in 24-hr culture supernatants of AM obtained by BAL (mean ± SE from at least three animals).
*Significantly greater than control, *p* < 0.05.

We used the first BAL sample (≈ 5 mL) to monitor chemokine protein and NO levels and performed ELISA on a small aliquot of the sample. IL-6 and MCP-1 levels were significantly elevated on day 1 at medium and high dose levels, but returned to basal levels by day 7 and remained at basal levels on day 30 (Figure 6). MIP-2 levels were elevated at all doses at the three time points studied (Figure 6). We found no change in NO levels in BAL fluid, as measured by the nitrite content, under any of the exposure conditions studied, but AMs cultured for 24 hr showed a significant increase in production of NO on day 1 for the two highest doses of DEPs (Figure 7). The NO production returned to normal levels by day 30; data were not collected for day 7.

### Inflammatory mediators in BAL cells cultured for 24 hr.

**Figure 8:**
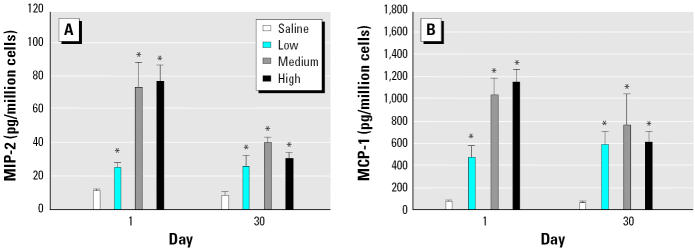
MIP-2 (*A*) and MCP-1 (*B*) production in 24-hr culture supernatants of AM obtained by BAL (mean ± SE from at least three animals).
*Significantly greater than control, *p* < 0.05.

AMs were cultured for 24 hr, and inflammatory mediator levels were measured in the culture supernatants. Both MIP-2 and MCP-1 protein levels were increased at all three doses of DEPs on days 1 and 30 after exposure (Figure 8). Data were not collected for day 7.

### *mRNA expression in* in vitro *co-culture experiments.*

Fibroblasts and AMs were co-cultured in Transwell chambers. We observed no change in the mRNA levels of IL-1β under any of the conditions tested (Table 1). Similarly, there was no change in iNOS mRNA expression under any conditions in the Transwell experiments. The data in Table 1 suggest that a main source of IL-6 and MCP-1 in the BAL fluid is the lung fibroblasts because they exhibit high message levels for these mediators compared with AMs. Whether there was an increase on co-culture with AMs and/or DEPs was difficult to assess because of extreme variability of the results.

## Discussion

AMs provide the first line of lung defense by eliminating most foreign material from distal airways (Sibille and Reynolds 1990). They play an important role in the initiation of inflammation and the acquired immune response by secreting a variety of cytokines (Fearon and Locksley 1996). Although AMs play a central role in this regard, lung parenchymal cells are also involved in the production of a number of important cytokines. In fact, in a previous study (Rao et al. 2004), we showed that lung fibroblasts are a major source of MCP-1 and IL-6 after silica exposure.

The aim of this study was to monitor the time course of mRNA expression of the genes implicated in the inflammatory response in BAL cells and the lung tissue after exposure to DEPs, and to correlate the message levels with the cytokine protein levels. iNOS activity was measured at the functional level (NO production). The mRNA expression in BAL cells was very different among the genes studied after *in vivo* DEP exposure. Four genes (*IL-1*β, *iNOS*, *MCP-1*, and *MIP-2*) were up-regulated within 24 hr after DEP exposure. Consistent with the observations at the message levels, the protein levels of MCP-1 and MIP-2 went up in BAL fluid within this time period. AM production of NO was increased 24 hr after DEP exposure. However, NO levels in BAL fluid were not elevated. NO has been proposed to play a wide variety of roles in macrophage function (Castranova et al. 1998; MacMicking et al. 1997; Mills 2001). Up-regulation of *IL-1*β, *iNOS*, and *MIP-2* genes in BAL cells by high DEP exposure was bimodal, with strong up-regulation at 1 and 30 days and basal levels at day 7. For *MCP-1*, the mRNA levels were significantly higher than control at all time periods for the two high doses of DEPs.

The expression of mRNA did not always correlate with the cytokine protein levels. Although MCP-1 mRNA levels stayed high throughout the experimental period, the BAL fluid cytokine levels were significantly elevated only on day 1. In the case of IL-6 the situation was reversed; there was a dose-dependent increase in cytokine levels in the BAL fluid on day 1 without a significant change in the mRNA levels. MIP-2 cytokine levels were elevated in the BAL fluid throughout the experimental period and at all dose levels, but message levels were up only on days 1 and 30 and only at high dose levels. These discrepancies may be related to the stabilities of the mRNA species and the proteins of the cytokines concerned and needs further investigation. In fact, posttranscriptional regulation is common among many inflammatory mediators (Kaspar and Gehrke 1994; Powell et al. 2000; Rao 1999).

MCP-1 plays an important role in the accumulation of monocytes (Leonard and Yoshimura 1990) and was up-regulated within 24 hr in BAL cells, at both the message and protein levels. Although an increase in MCP-1 mRNA levels was seen in BAL cells exposed *in vivo*, *in vitro* exposure of AMs to DEPs did not increase MCP-1 mRNA levels. Further, the *in vitro* studies reveal that lung fibroblasts are an important source of IL-6 and MCP-1. The increase in MCP-1 levels seen in the BAL fluid did not lead to an increase in the number of AMs observed in the BAL fluid at that time point. In contrast, the potent chemotactic factor for neutrophils, MIP-2 (Driscoll 1994), showed a direct correlation between levels in BAL fluid and PMNs yield by BAL. MIP-2 levels stayed high throughout the posttreatment period, with a corresponding elevation in PMNs harvested by BAL over this time.

Exposure to particulates such as asbestos, silica, and DEPs has been shown to up-regulate IL-1 levels (Hartmann et al. 1984; Schmidt et al. 1984; Yang et al. 1997). We show that IL-1β message level increases within 24 hr after DEP exposure *in vivo*. Although IL-1β is implicated in the induction of inflammation and cell recruitment, the up-regulation of *IL-1*β was relatively weak compared with that of *MCP-1* and *MIP-2*, both of which are potent chemoattractants.

IL-10 is an anti-inflammatory cytokine that inhibits cytokine production by AMs (Raychaudhuri et al. 2000) and human peripheral blood mononuclear cells (Wang et al. 1994). There was a no significant change in its expression in BAL cells after DEP exposure. Similarly, there was no change in another anti-inflammatory cytokine, *TGF-*β*1*.

Cytokine levels in lung tissue have been evaluated by other investigators after intratracheal instillation of DEPs (Takano et al. 2002). They found no significant change in IL-1β, ICAM-1, and MIP-1α mRNA levels in the lung tissue at 24 hr. We measured the expression of four other genes (*IL-6*, *GM-CSF*, *ICAM-1*, and *RANTES*) in lung tissue after lavage and found no change in the mRNA levels of these genes under any of the exposure conditions. Similarly, there was no increase in mRNA levels of *TNF-*α in BAL cells. Our observations concerning TNF-αmRNA level expression are consistent with observations that there is no increase in TNF-α at the protein level in AMs after *in vivo* (Yang et al. 2001) or *in vitro* DEP exposure (Yang et al. 1997).

In another study, cytokine levels were determined in AMs and lung tissue after inhalation exposure of DEPs for 1 month and 3 months in mice (Hiramatsu et al. 2003), which showed minimal changes in *TNF-*α, *IL-1*β, and *IL-10* expression. However, these researchers used gel electrophoresis to quantify the changes, which is much less sensitive than the real-time RT-PCR technique used in our study.

In summary, we have shown that exposure to DEPs *in vivo* causes a strong up-regulation of MCP-1, MIP-2, and iNOS mRNA within 24 hr, in a dose-dependent manner, in cells obtained by BAL. This is correlated with the appearance of MCP-1 and MIP-2 proteins in the BAL fluid. The up-regulation of iNOS message did not lead to a measurable increase of NO metabolic products in BAL fluid. However, supernatants of cultured AMs at 24 hr showed increased MCP-1 and MIP-2 levels, as well as increased NO production. There was a weak up-regulation of IL-1β mRNA levels. There was no increase in mRNA levels of IL-6, IL-10, TGF-β1, and TNF-α in BAL cells or IL-6, ICAM-1, RANTES, and GM-CSF in the lung tissue at any time point studied. Therefore, TNF-α does not seem to be involved in the pathologic changes associated with DEP exposure in the time period studied.

Results of the present studies, together with our previous observations concerning cytokine production after silica particle exposure (Rao et al. 2004), indicate that the early up-regulation of chemokines MCP-1 and MIP-2 is an important characteristic feature of particle-induced inflammatory response in the lungs. TNF-α seems to play no role in the initial exposure period. Further, our Transwell experiments reveal that *in vitro* stimulation of cells does not replicate the gene expression profile seen after *in vivo* exposure. We also performed some contact co-culture experiments with AMs and fibroblasts (data not shown), which showed results similar to those seen with the Transwell experiments. These observations support the concept that complex interactions between various cells and mediators within the lung microenvironment are involved in regulating the final inflammatory response. Our findings provide some clues to the mediators that are important in producing the changes associated with particle exposure that may help in designing informed intervention strategies.

## Figures and Tables

**Table 1:** Relative mRNA expression in AMs and lung fibroblasts (Fibro) stimulated with DEPs (200 μg/mL) for 4 hr in Transwell experiments.*^a^*

Insert/well	AMs*^b^*/None	None/Fibro*^b^*	AMs*^b^**/*Fibro	AMs/Fibro*^b^*	AMs*^b^* + DEPs*/*Fibro	AMs + DEPs*/*Fibro*^b^*	AMs*^b^**/*Fibro + DEPs	AMs/Fibro*^b^* + DEPs
IL-1β	1.07	0.18	2.7	0.62	2.81	0.83	3.34	0.17
IL-6	0.94	4,530	3.02	7,477	5.01	2,178	4.93	4,046
iNOS	0.86	0.11	2.98	0.31	2.79	0.23	3.29	0.25
MCP-1	0.95	88.54	4.09	340	4.14	119	2.84	248

a The numbers represent the average of two different experiments relative to mRNA levels in AM.

b Source of the cells in which the mRNA levels were measured.
